# Power outages and pediatric unintentional injury hospitalizations in New York State

**DOI:** 10.1097/EE9.0000000000000287

**Published:** 2023-12-14

**Authors:** Alexander J. Northrop, Nina M. Flores, Vivian Do, Perry E. Sheffield, Joan A. Casey

**Affiliations:** a Vagelos College of Physicians and Surgeons, Columbia University, New York; b Department of Environmental Medicine and Public Health, Icahn School of Medicine at Mount Sinai, New York City; c Department of Environmental Health Sciences, Mailman School of Public Health, Columbia University, New York; d Department of Environmental and Occupational Health Sciences, University of Washington School of Public Health, Seattle, Washington

## Abstract

**Background:**

In the past decade, electrical power disruptions (outages) have increased in the United States, especially those attributable to weather events. These outages have a range of health impacts but are largely unstudied in children. Here, we investigated the association between outages and unintentional injury hospitalizations, a leading cause of childhood morbidity.

**Methods:**

The study setting was New York State (NYS) from 2017 to 2020. Outage exposure was defined as ≥10%, ≥20%, and ≥50% of customers from a power operating locality without power, ascertained from NYS Department of Public Service records and stratified by rural, urban non-New York City (NYC), and NYC regions. Outcome daily block group-level pediatric injury hospitalization data was from the Statewide Planning and Research Cooperative System (SPARCS). We leveraged a case-crossover study design with logistic conditional regression.

**Results:**

We identified 23,093 unintentional injury hospitalizations in children <18 years with complete block group and exposure data. Most hospitalizations occurred in urban regions (90%), whereas outages were more likely in rural than urban areas. In urban non-NYC regions, outages ≥4 hours were associated with 30% increased odds of all-cause unintentional injury hospitalizations when ≥50% of customers were without power. Analyses by injury subtype revealed increasing point estimates as the proportion of customers exposed increased. These results, however, had wide confidence intervals.

**Conclusions:**

Outage exposure differed significantly across rural, urban non-NYC, and NYC regions across New York. Especially at the highest outage threshold, we observed an increased risk of pediatric unintentional injury hospitalizations.

**What this study adds**

To our knowledge, no research in the United States has specifically evaluated the effect of power outages as a primary exposure on children’s health. In this New York State-based study, we conceptualized an exposure metric for power outages, demonstrated differences in power outage exposure across urbanicity, and observed an increase in pediatric unintentional injury hospitalizations in the urban regions of New York during the most severe power outage exposures. Thus, this study (1) provides a framework for studying the effect of power outage exposure on health outcomes and (2) demonstrates that children’s health may be deleteriously affected by power outages.

## Introduction

Since 2010, electrical power disruptions (outages) have increased in the United States, especially those attributable to major weather events.^1^ Climate change is projected to increase electric energy demand,^2^ straining an aging infrastructure system that will be further impacted by extreme weather events.^3^ Already, over 60% of outages nationwide are attributable to extreme weather events.^4^ While weather events affect energy infrastructure, access to reliable and affordable energy, or energy security, is also closely intertwined with individuals’ vulnerability to climate change.^5^

Increasingly, these outage events have been linked to various adverse health effects, including temperature-related illness,^6^ increased rates of carbon monoxide poisoning,^7,8^ and disruption of electricity-dependent durable medical equipment.^9^ However, consistent with the dearth of research on pediatric-specific environmental health outcomes, few population-level studies evaluate the association between outages and children’s health.^10^ While a single-center study in Cape Town, South Africa, found a 10% increase in pediatric admissions during outages,^11^ no similar study has been conducted elsewhere, including the United States. With an increasingly vulnerable electrical distribution system, characterization of the downstream health effects of outages can inform primary and secondary prevention efforts.

Of particular concern is the effect of outages on injury hospitalizations, the leading cause of hospitalizations for children in the United States.^12^ Injury hospitalizations include burns, drownings, falls, environmental exposures, lacerations, transportation injuries, poisonings, firearm injuries, overexertion, and suffocations. Extreme weather events that co-occur with outages, such as anomalously warm temperatures, have been projected to increase injury deaths across the nation.^4,13^ Several studies have described increases in injuries during severe weather events with co-occurring outages, such as cyclones, but isolating outages as a driver of these associations has proven difficult.^10,14–18^ Most analyses of outages and health outcomes leverage coarse (e.g., city-wide) spatiotemporal data or focus on single outage events, limiting the precision and generalizability of results.^10^

An additional challenge in assessing the impact of outage events on health is ascertaining the exposure given significant differences in electricity distribution—such as overhead versus underground transmission lines—and energy use^19^ across the urbanicity spectrum. Moreover, evidence from outage data at the county level^20^ and outside the conterminous United States suggests urbanicity affects the prevalence of outages and restoration times.^21^

Here, we leverage highly spatially resolved outage data in New York State (NYS) to quantify multi-year sub-county outage exposures among rural, urban non-New York City (NYC), and NYC regions of the state. Then, we estimate the association between power outages and pediatric unintentional injury hospitalizations across these geographic strata.

## Methods

### Study population

We used a case-crossover study design with hospitalization data from the Statewide Planning and Research Cooperative System (SPARCS). The SPARCS database is a comprehensive reporting system that includes patient-level information of all hospitalizations in New York State. Cases included children aged <18 years admitted to a New York hospital from January 2017 to December 2020 for unintentional injury. Hospitalization visits represent less frequent and more severe injury outcomes than most emergency department visits.

The Mount Sinai Institutional Review Board approved the study (#19-00355).

### Exposure classification

The NYS Department of Public Service provided outage data at power operating localities (POL) (N = 1,865), the finest geographic resolution of outage data reported to the state. These data exist in 30-minute intervals with the number of customers served and the number of customers without power for each interval. Customers represent end-users for electric utility companies, including single-family homes, apartments, and businesses. We excluded POLs with <30 customers or >5% temporal missingness over the study period, resulting in 1,764 (94.6%) included POLs. We defined outages at the POL level using a 36-hour cumulative metric, aggregating hours where ≥10%, ≥20%, or ≥50% of customers were without power immediately preceding admission. We selected a 36-hour cumulative exposure metric after hypothesizing that outage events would impact acute hospitalization rates.

The patient’s residential location was available based on the geocoded block group. As such, we assigned outage exposure to patients based on the POL within which their block group population centroid was located.

### Outcome assessment

From the International Classification of Diseases 10 (ICD-10) external cause-of-injury framework developed by the National Center for Health Statistics and National Center for Injury Prevention and Control,^22^ ICD-10 codes were matched to 15 injury categories. These groupings provide a standardized approach to categorizing injuries that have previously been reported in the literature.^23,24^ A recent single-center study by Clery et al.^25^ found that ICD-10 clinical modification injury codes had a sensitivity of 67% and a specificity of 90% for identifying injuries at large. Notably, these were provider-assigned codes in an emergency department as opposed to the hospitalization claims data used in SPARCS.^25^ The following injury categories were included in analyses: cut/pierce, drowning, fall, fire/burn, environmental, motor-vehicle (traffic), transportation (other), poisoning (drug), poisoning (non-drug), overexertion, machinery, firearm, struck by/against, suffocation, and other. We excluded unspecified injuries from the analysis.

External cause ICD-10 codes from the matrix could be contained within the primary, admitting, external cause of injury, or “other” diagnoses. Each hospitalization record contained exactly one primary and admitting diagnosis. The number of other and external cause of injury diagnoses varied among the records (eTable 1; http://links.lww.com/EE/A255). We included all available SPARCS diagnoses linked to hospitalization, including primary and associated diagnoses, in the analysis. However, to ensure that only one contributing diagnosis was given to each unique admission record, we instituted a series of rules to ensure that the external code used was as closely aligned with the cause of admission as possible.

For any instance of multiple injury external codes, we used the most specific code; that is, when two external codes were associated with an admission record (e.g., transport and other), we used the nonother (e.g., transport) category. Due to the high number of admission records with both poisoning and fire/burn categories, we decided to attribute these records to fire/burn, since most of the records involved smoke inhalation and related diagnoses, which are closely related to fire exposure. Additionally, we combined several closely related fields: motor vehicle (traffic) and transportation (other) to create the transport category, as well as poisoning (drug) and poisoning (nondrug) to create the poisoning category. When these rules failed to parse a single external code associated with an admission record, we created the category “multiple” to designate an injury category for the admission record. Ultimately, 14 external cause of injury categories were used for the analyses.

### Covariate assessment

For meteorologic covariates, we used Parameter-elevation Regressions on Independent Slopes Model (PRISM) data.^26^ Specifically, we utilized daily cumulative precipitation and mean temperature at the block group level. Since PRISM data is aggregated at the daily time scale, with days defined to start at noon coordinated universal time , we transformed the hourly SPARCS data and outage data to align with the PRISM-defined day.

To address differences in outage exposure by urbanicity, we used 2010 US Census population data at block group level^27^ to assign POLs an urbanicity classification using urban/rural population values with areal interpolation.^28^ POLs with >50% of rural inhabitants were classified as rural; otherwise, they were assigned as urban.^29^ NYC classification was based on county boundaries (Bronx, Kings, New York, Queens, and Richmond counties). These rules resulted in the following categories: rural (N = 985, 55.8%), urban non-NYC (N = 703, 39.9%), and NYC (N = 76, 4.3%).

### Statistical analysis

We conducted descriptive analyses and used Kruskall–Wallis and Wilcoxon Ranked Sum tests to compare average exposure duration by urbanicity.

We used a case-crossover study design to investigate the relationship between outages and pediatric injury hospitalizations,^30,31^ matching hospitalization records with exposure data on the hour of admission, day of week, month, and year. For all-cause injury analyses, we stratified models by urbanicity. However, for injury subgroup analyses, we pooled cases across urbanicity types due to low counts of hospitalizations. We used conditional logistic regression models^30^ and controlled for daily cumulative precipitation linearly and mean temperature nonlinearly using three knots (eFigure 1; http://links.lww.com/EE/A255).

We conducted several sensitivity analyses to assess the robustness of our study. To assess the impact of the COVID-19 pandemic on our results, we re-ran the analyses, excluding all 2020 data. Additionally, we used a one percent of customers power outage threshold to assess the impact of smaller-scale outages. These results informed if lower thresholds would be needed to study the effects of power outages in densely populated areas, such as NYC, where a lower percentage of customers tend to be without power at a given time. Finally, we assessed the impact of the exposure window (i.e., 12 hours, 24 hours, 36 hours, or 48 hours before hospitalization) on the results.

We conducted all analyses using R (version 3.6.3; version 4.2.2, The R Foundation for Statistical Computing, Vienna, Austria)^32^ and the *survival* package.^33^

## Results

Between 2017 and 2020, the average NYS customer experienced 25.6 hours without power (7.8 million NYS customers total and 200 million customer hours without power). Most customers were in urban non-NYC POLs (N = 3,686,060 [47%]) followed by NYC POLs (N = 3,006,017 [38%]) and rural POLs (N = 1,157,790 [15%]) (eFigure 2; http://links.lww.com/EE/A255). Most customer-hours without power were in urban non-NYC POLs (127 million customer-hours [63%]), followed by rural POLs (60 million customer-hours [30%]), and NYC POLs (15 million customer-hours [7%]).

**Figure 1.**
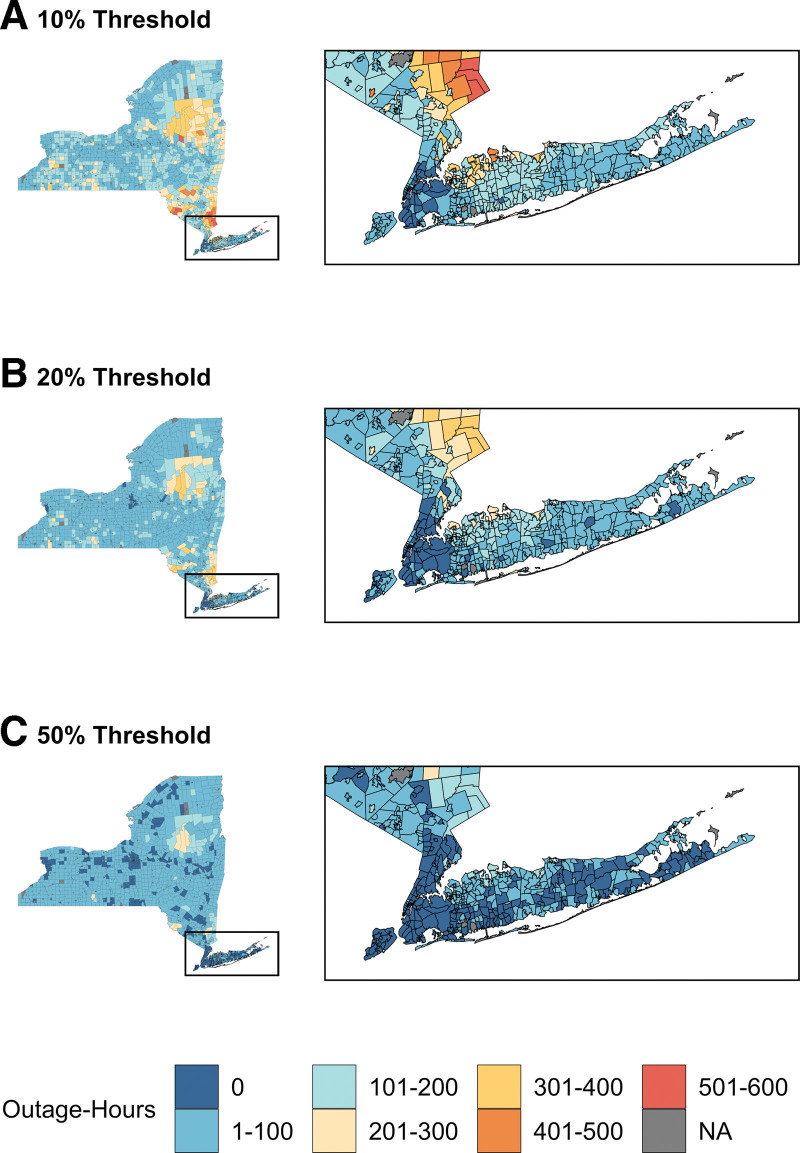
Outage-hours per POL from 2017 to 2020. Number of outage hours per power operating locality (POL) within New York State from 2017 to 2020.

Of these outages, there were 175,286 POL-hours ≥10% threshold across New York State, which corresponded to over 137 million customer-hours without power. This metric captures 68% of all customer hours with outages (eTable 2; http://links.lww.com/EE/A255). On average, rural POLs experienced higher median yearly outage hours (22 hours; IQR 12–37; maximum: 134) than urban non-NYC (18 hours; IQR 7–32; maximum: 132) or NYC POLs (0 hours; IQR 0–1; maximum: 24) at the ≥10% threshold. Figure 1 shows the distribution of these outage hours without power at the ≥10%, ≥20%, and ≥50% thresholds across New York.

**Figure 2.**
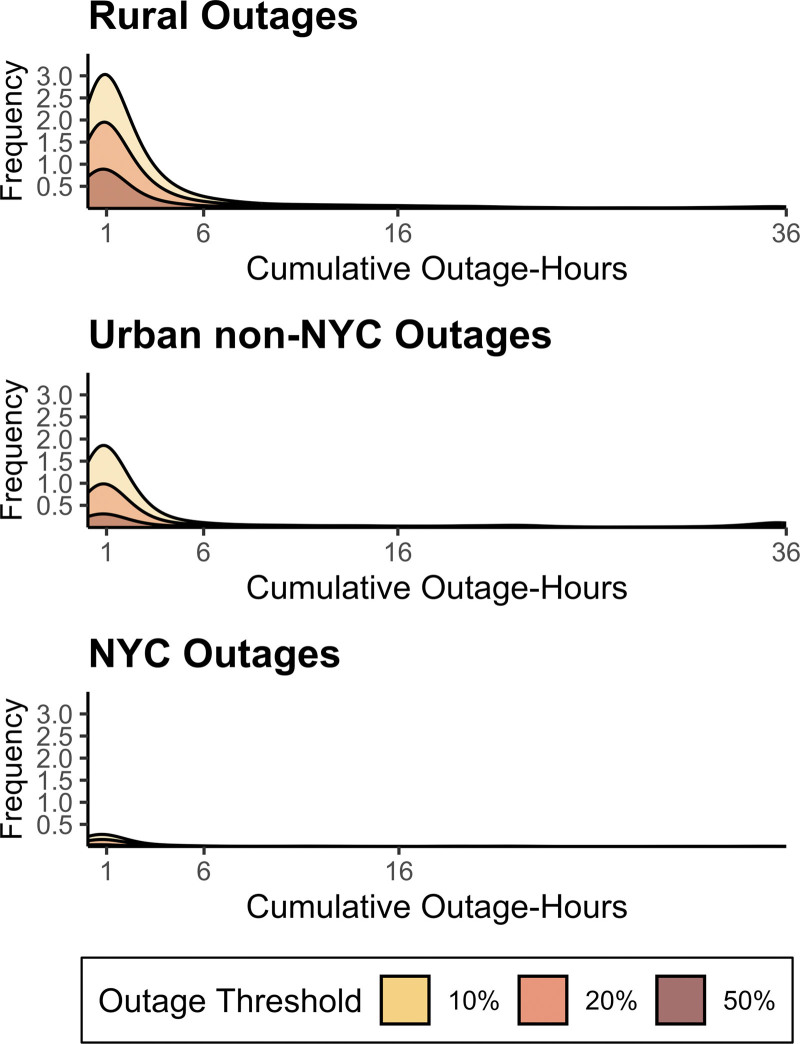
Outage-hour dstributions across the geographic strata. The y-axis, “Frequency,” represents the number of days in a year a power operating locality (POL) experiences an outage using the 36-hour cumulative metric as a density curve. The x-axis represents the cumulative hours of outages POLs experience on all days with at least one outage hour. For example, over the 4-year study period, rural POLs averaged <1 day for an outage impacting ≥50% of customers >1 hour.

When evaluating the 36-hour metric, most outages were relatively short. Across all geographic strata and among all days with at least one outage hour, POLs experienced a median of 1.2 hours (IQR: 0.5–3.0) without power in the 36 hours preceding injury hospitalizations (Figure 2). Outage periods were slightly longer in rural regions (median: 1.2 hours; IQR: 0.6–3.0; *P* < 0.01) than in urban regions (median: 1.0; IQR: 0.5–3.0; *P* < 0.01) and the NYC region (median: 1.0; IQR: 0.4–2.0; *P* < 0.01).

**Figure 3.**
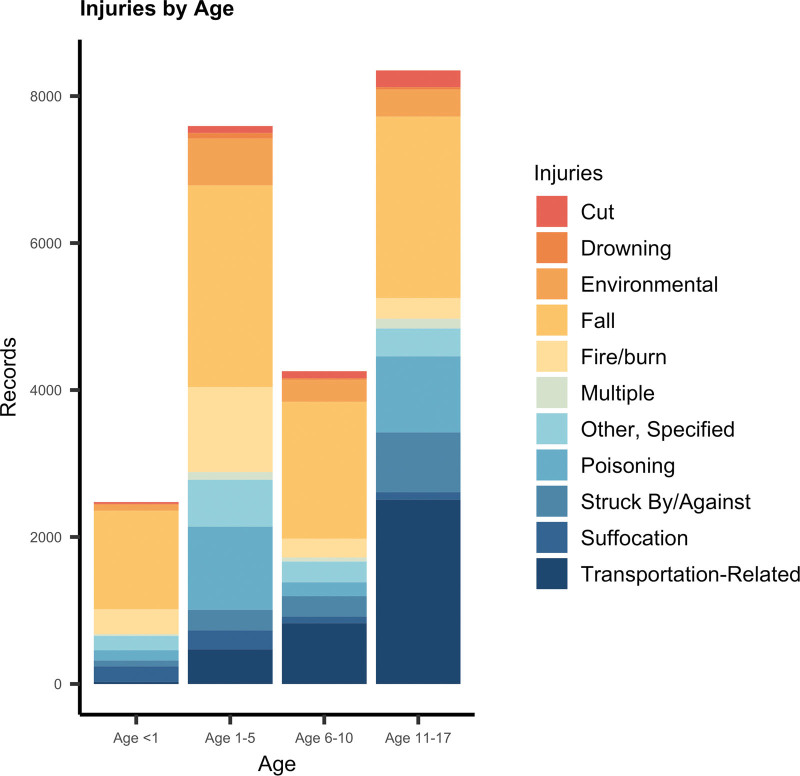
Injuries by age in New York State from 2017 to 2020. Several categories were excluded for low cell counts, including machinery, firearm, and overexertion categories.

Regarding the outcome of interest, we identified 30,978 unintentional injury hospitalization records in NYS from January 2017 to December 2020, of which 25,214 had a specified injury diagnosis (81.4%) and 23,093 (74.5%) records had complete block group and power outage data (eFigure 3; http://links.lww.com/EE/A255). The majority of injuries during the study period were falls (N = 8,422), and transportation-related injuries (N = 3,835) with the incidence of transportation-related injuries increasing with increased age (Figure 3). Moreover, there were clear geographic trends in the subcategories contributing to unintentional injury hospitalizations: falls comprised 40% of unintentional injury hospitalizations in NYC but only 23% in rural regions. In contrast, transportation-related admissions comprised a larger percentage of hospitalizations in rural areas (28%) versus NYC (13%) (eTable 3; http://links.lww.com/EE/A255). From 2017 to 2020 in NYS, there were more male unintentional injury hospitalizations (60%) than female unintentional injury hospitalizations (40%) (eFigure 4; http://links.lww.com/EE/A255).

Most hospitalizations (N = 20,880, 90.4%) occurred among children residing in urban regions of the state (Table 1). One hundred sixty-nine hospitalizations co-occurred with an outage event in the preceding 36 hours from admission. While this number is low, this also reflects the relative rarity of pediatric hospitalization; there were an average of 16 unintentional injury hospitalizations per day across NYS during the study period.

**Table 1.** Unintentional injury records demographic information

Age	Age <1	Age 1 - ≤5	Age 6 - ≤10	Age 11 - ≤17	All Ages
Total Cases	n = 2484	n = 7654	n = 4292	n = 8663	n = 23093
	No. (%)	No. (%)	No. (%)	No. (%)	
Sex					
Female	1074 (43)	3236 (42)	1708 (40)	3111 (36)	9129
Male	1409 (57)	4418 (58)	2584 (60)	5550 (64)	13961
Race and ethnicity					
Asian	167 (7)	468 (6)	266 (6)	409 (5)	1310
Black	430 (17)	1377 (18)	764 (18)	1638 (19)	4209
Hispanic	439 (18)	1500 (20)	813 (19)	1553 (18)	4305
Multi-ethnic and/or multi-racial	64 (3)	166 (2)	80 (2)	115 (1)	425
Other	424 (17)	1197 (16)	590 (14)	996 (11)	3207
White	960 (39)	2946 (38)	1779 (41)	3952 (46)	9637
Season of injury^b^					
Winter	584 (24)	1515 (20)	726 (17)	1793 (21)	4618
Spring	596 (24)	1941 (25)	1193 (28)	2109 (24)	5839
Summer	683 (27)	2406 (31)	1477 (34)	2664 (31)	7230
Fall	621 (25)	1792 (23)	896 (21)	2097 (24)	5406
Year of injury					
2017	614 (25)	2241 (29)	1246 (29)	2377 (27)	6478
2018	661 (27)	1961 (26)	1064 (25)	2179 (25)	5865
2019	678 (27)	1902 (25)	1061 (25)	2227 (26)	5868
2020	531 (21)	1550 (20)	921 (21)	1880 (22)	4882
Cause of injury^c^					
Cut	24 (1)	95 (1)	96 (2)	229 (3)	444
Drowning	13 (1)	77 (1)	21 (0)	33 (0)	144
Fall	1341 (54)	2745 (36)	1863 (43)	2473 (29)	8422
Fire/burn	338 (14)	1158 (15)	257 (6)	281 (3)	2034
Multiple	26 (1)	104 (1)	54 (1)	132 (2)	316
Environmental	82 (3)	637 (8)	299 (7)	366 (4)	1384
Transportation-related	29 (1)	472 (6)	827 (19)	2507 (29)	3835
Poisoning	143 (6)	1132 (15)	189 (4)	1035 (12)	2499
Struck by/against	77 (3)	280 (4)	279 (7)	813 (9)	1449
Suffocation	212 (9)	257 (3)	90 (2)	104 (1)	663
Other, specified	191 (8)	663 (9)	281 (7)	378 (4)	1513
Length of hospital stay (median days)	2	1	1	2	2
Urbanicity					
Rural	146 (6)	631 (8)	426 (10)	1010 (12)	2213
Urban non-NYC	1089 (44)	3163 (41)	1815 (42)	4088 (47)	10155
NYC	1249 (50)	3860 (50)	2051 (48)	3565 (41)	10725

Percentages may not sum to 100% due to rounding and/or excluded cells.

a The unknown sex category was excluded for low cell counts <10.

b Seasons were defined based on yearly solstices and equinoxes (i.e., Summer 2019: June 21–September 22).

c Several categories were excluded for low cell counts, including machinery, firearm, and overexertion.

NYC, New York City.

**Figure 4.**
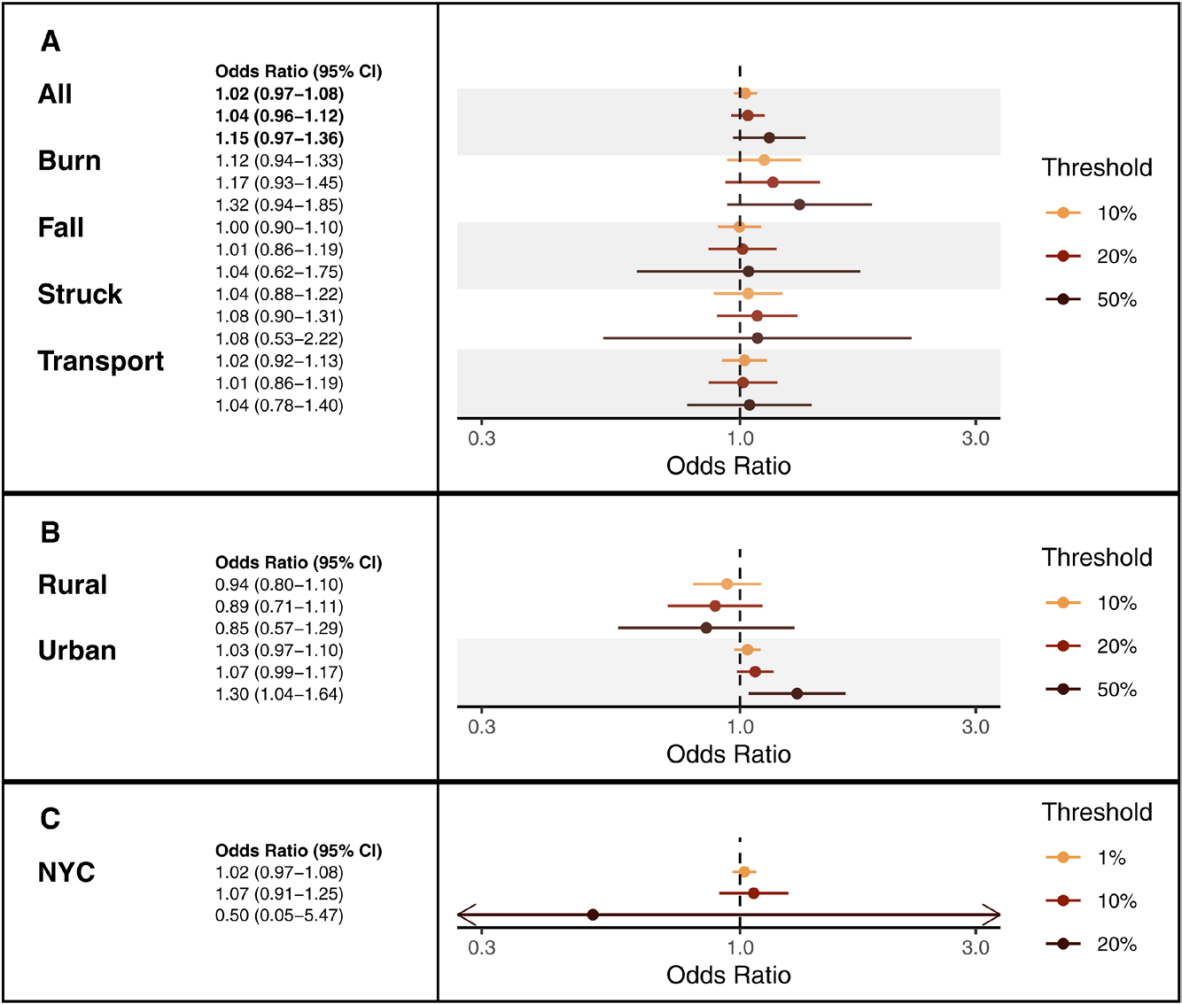
The association between power outages and pediatric injury hospitalizations in New York State, 2017–2020. Odds ratios and 95% confidence intervals for injury subgroups with sufficient data (i.e., sufficient co-occurring incidents of injury hospitalizations and power outage exposure) are included in *Panel A. Panel B* includes all-cause unintentional injuries for rural and urban non-New York city (NYC) regions of New York State. *Panel C* includes all-cause unintentional injuries for NYC. Thresholds for *Panels A and B* refer to the operationalized power outage threshold of ≥10%, ≥20%, and ≥50% of customers without power to define an outage-hour. *Panel C* features thresholds of ≥1%, ≥10%, and ≥20% of customers without power to reflect the infrequency of ≥50% threshold exposure in NYC.

Pooled subgroup analyses revealed increased effect estimates with increased outage thresholds (i.e., the proportion of customers exposed) for all subcategories with sufficient counts for the models, but confidence intervals (CI) were wide (Figure 4).

In urban non-NYC POLs, outages were associated with increased all-cause unintentional injury hospitalizations at the ≥50% threshold. For every 4 hours with ≥50% of customers in a non-NYC urban POL without power in a 36-hour period, there was an associated 30% increase in the odds of a child being hospitalized for an unintentional injury. In contrast, rural and NYC outages were not associated with all-cause unintentional injury hospitalizations. In fact, while we observed a trend of increased point estimates for increased outage intensity across the pooled sub-categories and urban strata, these trends did not persist for rural unintentional injuries. Instead, effect estimates decreased for increased outage intensity in the rural stratum. These results were consistent when only considering hospitalizations from 2017 to 2019, eliminating the effect of the COVID-19 pandemic on the results (eTable 4; http://links.lww.com/EE/A255).

To elucidate the impact of smaller-scale outages that may not have been captured with the predefined outage thresholds, we ran a sensitivity analysis with a 1% threshold for power outages across all regions (eTable 5; http://links.lww.com/EE/A255). Using a 1% threshold, power outages were associated with all-cause unintentional injury across all regions (OR = 1.02; 95% CI = 1.00, 1.05) and by urbanicity type (rural OR = 1.03; 95% CI = 0.95, 1.13; urban non-NYC OR = 1.02; 95% CI = 0.99, 1.06; NYC OR = 1.02; 95% CI = 0.97, 1.08). Across all regions using the 1% threshold, we observed elevated odds of fire-related (OR = 1.09; 95% CI = 1.00, 1.19) transport-related (OR = 1.05; 95% CI = 0.99, 1.11), and overexertion (OR = 1.44; 95% CI = 0.90, 2.31) injury hospitalizations.

We also evaluated the influence of the selected exposure window (i.e., the period of time before the admission) on model results. Generally, the results remained consistent. We did observe, however, that shorter exposure windows frequently led to larger odds ratios (eTable 6; http://links.lww.com/EE/A255). For example, non-NYC urban pediatric unintentional injuries (all types combined) had larger odds ratios at the 12-hour (OR = 1.88; 95% CI = 1.05, 3.36) and 24-hour (OR = 1.52; 95% CI = 1.08, 2.15) exposure windows compared with the 36-hour window used in the main analysis at the ≥50% customer outage threshold.

## Discussion

In this multi-year study of pediatric unintentional injury hospitalizations across NYS, we found an association between electrical power outages and increased all-cause unintentional injury hospitalizations in urban non-NYC regions. This association strengthened as the percentage of customers without power increased. When the power goes out, children may have increased injury risk associated with extreme temperatures, gas-powered generators,^7^ open flames, and dimly lit spaces in urban non-NYC regions of NYS.

While prior studies have incorporated NYS POLs in health analyses,^34,35^ we uniquely stratified according to urbanicity, which revealed different patterns of exposure and outcomes. Urban regions of the state, while experiencing fewer outages than rural regions of the state, comprise over 90% of the pediatric injury hospitalizations in the state. Hence, these findings may be driven by our ability to detect the association between outages and unintentional injuries in urban non-NYC POLs that we could not elsewhere in the state. These results may also suggest that urban environments are more dangerous during outages, a finding consistent with recent literature.^36^ In contrast, while the 95% CIs were wide, children in rural POLs appear to have fewer hospitalizations with a higher exposure intensity. This pattern may indicate decreased healthcare-seeking behavior during outages or differences in relative injury incidence across urbanicity strata

With a low frequency of exposure, NYC outages at the 10%, 20%, and 50% thresholds did not appear to affect unintentional injury hospitalizations. These findings highlight the difficulties in assessing the effect of outages in densely populated regions or in geographic units with high population counts. Although outages are less frequent in NYC compared with the rest of the state (15 million customer outage hours versus 127 million urban non-NYC and 60 million rural customer outage hours), a substantial proportion of outages in NYC did not meet the threshold for inclusion as an outage (eTable 2; http://links.lww.com/EE/A255). In our primary analysis, we used a minimum outage threshold of 10% of customers outage. The threshold was infrequently exceeded in NYC, likely due to the high population density. We did observe a 2% increase in the odds of injury hospitalizations using a lower 1% customer outage threshold. Further, across the state, we observed evidence of an association with burn, transport, and overexertion injuries at the 1% outage threshold. This suggests that these smaller-scale outages may have important health implications, consistent with prior research on outage exposures that incorporated lower (0.5%) thresholds.^34^

While we did not incorporate lagged effects in our main model given the hypothesized acute effects of outages on injury hospitalizations, sensitivity analyses suggested that the largest impact on hospitalizations occurred 12 to 24 hours before hospital admission. Future research may incorporate these parameters to inform the selection of exposure windows.

Our study had several strengths. We included comprehensive statewide hospitalization data and applied a study design that controls for time-invariant confounders. However, our study also had limitations. Because we defined outages based on a single location and POL of residence, exposure misclassification is possible. Children who represented our cases may not have been at their residence during the power outage events and, even if they were, their homes may not have been directly impacted. Exposure data with a higher geospatial resolution, such as satellite imagery,^37^ or household-level reports of outages, would support the detection of the health impacts of outages in densely populated areas. Injury hospitalizations also represent an extreme health outcome related to outages and likely underestimate the impact of outages on unintentional pediatric injuries. Nevertheless, pediatric hospitalizations represent a significant emotional and physical impact on society. These hospitalizations also represent significant financial costs: on average, one pediatric hospitalization costs upward of $10,000.^38^ While severe injuries resulting in out-of-hospital and emergency department mortality would not be captured in our analyses, these events are rare. Prehospitalization mortality occurred in fewer than 2% of total pediatric injury cases at a high-volume level 1 trauma center, indicating that pediatric hospitalizations capture the bulk of severe injury outcomes.^39^

Future analyses should include more years, lagged effects, larger geographic scope, and/or emergency department and outpatient data to enhance our understanding and identify points of intervention to mitigate power outages, a growing pediatric health threat, in a changing climate. Child health and outage research can also help inform and further motivate the modernization of the US electrical grid, which is under pressure from both increasingly extreme weather and an increasing demand for electrification.

## Conclusion

Outage exposure differs across rural, urban non-NYC, and NYC regions across New York. Especially at the highest outage threshold, we observed an increased risk of pediatric unintentional injury hospitalizations in urban regions of New York, excluding New York City. The effect estimate generally grew with decreased exposure windows and increased exposure intensity. These results reveal a potential relationship between power outages and unintentional injuries among children. Further study of power outages and emergency department visit injuries may allow further exploration of threshold effects and inform recommendations for preventative action.

## Supplementary Material
