# Supplementary material for: Power outages and pediatric unintentional injury hospitalizations in New York State
Source: Environ Epidemiol. 2023 Dec 14;8(1):e287. doi: 10.1097/EE9.0000000000000287 (PMC10852386; doi:10.1097/EE9.0000000000000287)
Supplement: Supplementary file 1 [file ee9-8-e287-s001.pdf]

SUPPLEMENTARY MATERIALS

eFigure 1: Causal Directed Acyclic Diagram

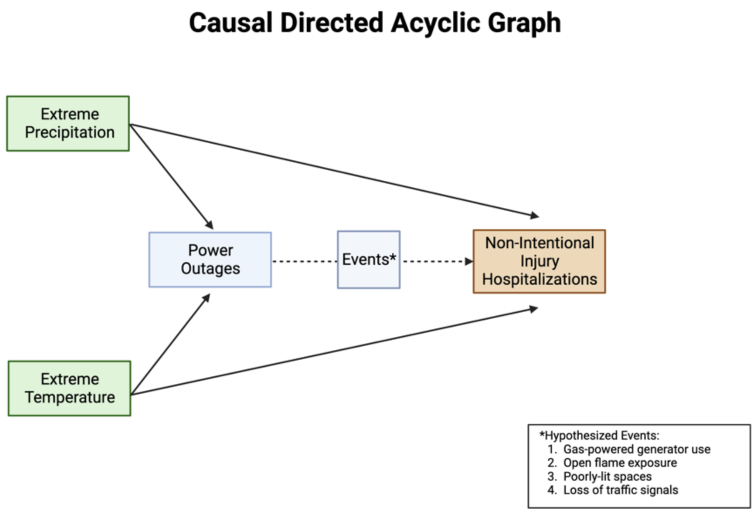

eFigure 2: Urbanicity Classification by Power Operating Locality

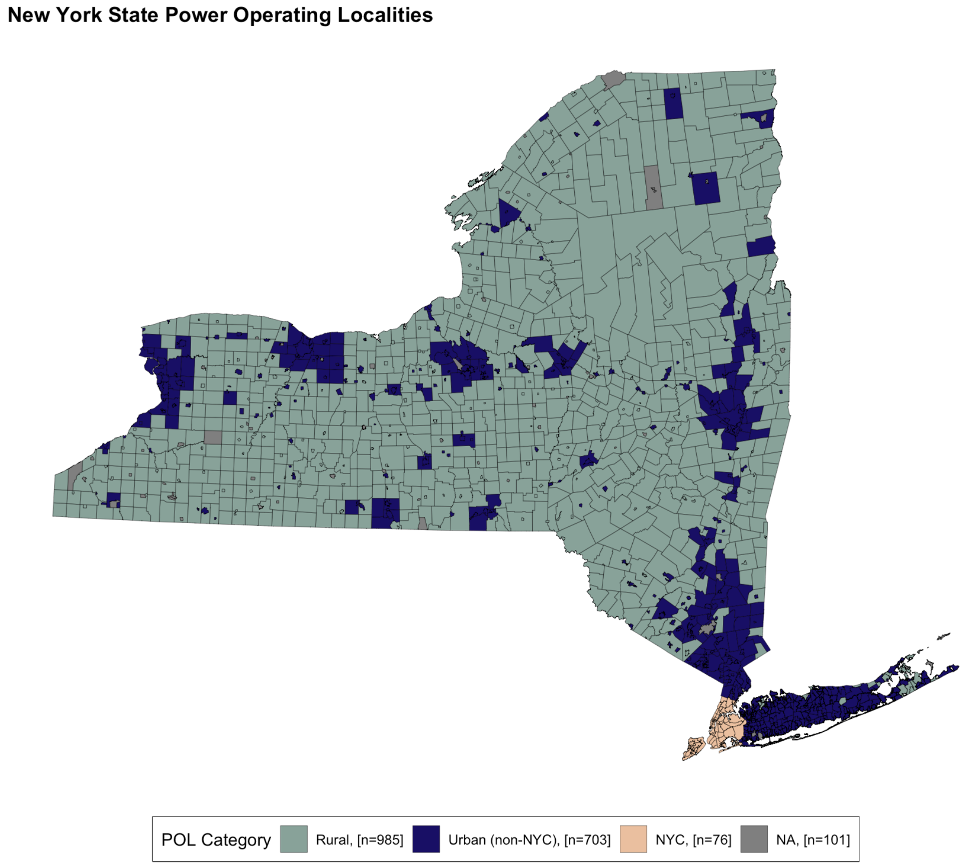

eFigure 3: Inclusion Flow Chart

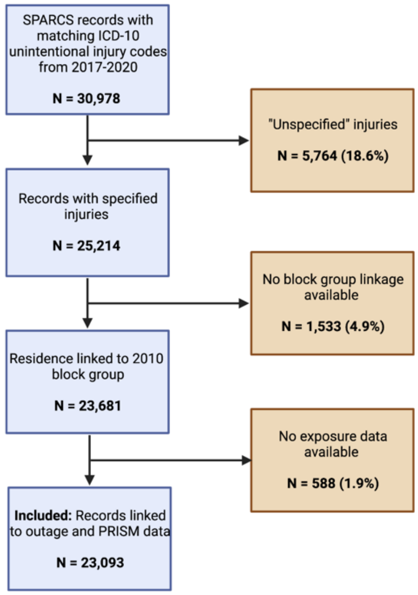

eFigure 4: Unintentional Injury Records by Sex

Records from the SPARCS database from 2017-2020 in New York State.

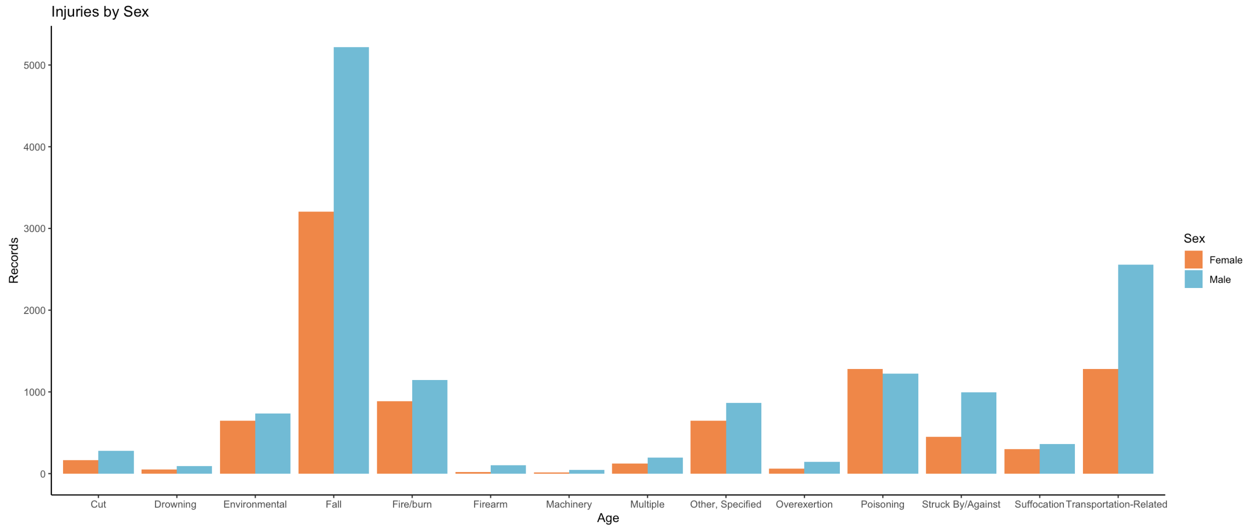

eTable 1: Diagnoses per SPARCS Hospitalization Record

The number of primary, admitting, "other," and external cause diagnoses among all pediatric hospitalizations (age <18) from 2017-2020 in the SPARCS database.

Diagnosis Category	Minimum	1st Quartile	Median	Mean	3rd Quartile	Max
**Primary**	1	1	1	1	1	1
**Admitting**	1	1	1	1	1	1
**Other**	0	1	3	3.4	4	24
**External Cause**	0	0	0	0.087	0	9

eTable 2: Customer-Outage-Hours without Power

The number of customer-outage-hours without power captured by the ≥10% metric and total customer-outage-hours

Strata	**Customer-Outage-Hours** Incorporated in ≥10% Metric (% Total)	**Customer-Outage-Hours** Total (% All)
**Rural**	46,246,317 (78)	59,658,238 (30)
**Urban (non-NYC)**	87,849,243 (69)	126,605,040 (63)
**NYC**	3,423,656 (23)	14,691,736 (7)
**All**	137,519,216 (68)	200,955,014

eTable 3: Unintentional Injury Hospitalizations by Urbanicity

The number of pediatric unintentional injury hospitalizations by external cause-of-injury framework grouping from 2017-2020 in the SPARCS database. Note, the categories firearm and machinery were excluded due to low cell counts.

Cause of Injury	Rural	Urban non-NYC	NYC	Statewide
**All**	2213	10155	10725	23093
	No. (%)	No. (%)	No. (%)	No. (%)
**Cut**	58 (3)	184 (2)	202 (2)	444 (2)
**Drowning**	22 (1)	82 (1)	40 (0)	144 (1)
**Fall**	500 (23)	3534 (35)	4288 (40)	8322 (36)
**Fire/Burn**	126 (6)	776 (8)	1132 (11)	2034 (9)
**Multiple**	36 (2)	115 (1)	165 (2)	316 (1)
**Environmental**	148 (7)	585 (6)	651 (6)	1384 (6)
**Transportation-Related**	626 (28)	1847 (18)	1362 (13)	3835 (17)

**Poisoning**	216 (10)	1192 (12)	1091 (10)	2499 (11)
**Struck By/Against**	142 (6)	702 (7)	605 (6)	1449 (6)
**Suffocation**	49 (2)	327 (3)	287 (3)	663 (3)
**Other, Specified**	136 (6)	638 (6)	739 (7)	1513 (7)
**Overexertion**	22 (1)	86 (1)	97 (1)	205 (1)

eTable 4: Conditional Logistic Regression Model Results, 2017-2019

The conditional logistic regression model was re-run only matching records from 2017 to 2019 in order to eliminate the effect the COVID-19 pandemic on the results. Cells in grey represent NA results due to low co-occurring exposure and outcome events. Lower and upper represent the lower and upper bounds of the effect estimate at the 95th percent confidence interval.

Strata	Threshold	Subgroup	OR	Lower	Upper
**Rural**	10	All	1.04	0.88	1.23
**Urban**	10	All	1.10	0.98	1.24
**NYC**	10	All	1.11	0.52	2.36
**Rural**	20	All	0.99	0.78	1.24
**Urban**	20	All	1.11	0.95	1.29
**NYC**	20	All	0.97	0.06	16.01
**Rural**	50	All	0.95	0.61	1.48
**Urban**	50	All	1.36	0.99	1.86
**NYC**	50	All			
**All**	10	All	1.08	0.99	1.19
**All**	20	All	1.07	0.94	1.21
**All**	50	All	1.20	0.96	1.49
**All**	10	Burn	1.21	0.92	1.58
**All**	20	Burn	1.27	0.93	1.72
**All**	50	Burn	1.54	0.88	2.70
**All**	10	Transport	0.99	0.74	1.33

**All**	20	Transport	0.48	0.14	1.63
**All**	50	Transport			
**All**	10	Struck	1.13	0.90	1.42
**All**	20	Struck	1.12	0.86	1.47
**All**	50	Struck	1.18	0.56	2.50
**All**	10	Fall	1.03	0.86	1.24
**All**	20	Fall	0.96	0.73	1.27
**All**	50	Fall	0.70	0.26	1.91

eTable 5: Conditional Logistic Regression Model Results, 1% Threshold

The conditional logistic regression model results using a 1% threshold. Lower and upper represent the lower and upper bounds of the effect estimate at the 95th percent confidence interval.

Strata	Subgroup	OR	Lower	Upper
Rural	All	1.03	0.95	1.13
Urban	All	1.02	0.99	1.06
NYC	All	1.02	0.97	1.08
All	All	1.02	1.00	1.05
All	Fire	1.09	1.00	1.19
All	Transport	1.05	0.99	1.11
All	Poisoning	1.04	0.95	1.13
All	Environment	0.89	0.75	1.05
All	Struck	1.00	0.89	1.12
All	Suffocation	0.82	0.59	1.14
All	Multiple	1.05	0.80	1.37
All	Other	0.98	0.87	1.11
All	Fall	1.01	0.96	1.06
All	Overexertion	1.44	0.90	2.31

All	Drown	0.90	0.60	1.35
All	Firearm	0.77	0.28	2.08
All	Machinery	1.42	0.82	2.44
All	Cut	1.10	0.91	1.33

eTable 6: Conditional Logistic Regression Model Results, 12-, 24-, and 48-Hour Exposure Windows
The conditional logistic regression model results using 12-, 24-, and 48-hour exposure windows. Lower and upper represent the lower and upper bounds of the effect estimate at the 95th percent confidence interval. Table A represents the rural strata, B the urban non-NYC strata, C the NYC strata, and D the pooled results

A. Rural Strata

Strata	Threshold (%)	Subgroup	OR	Lower	Upper	Window (hours)
Rural	10	All	0.83	0.57	1.21	12
Rural	10	All	0.89	0.70	1.13	24
Rural	10	All	0.95	0.83	1.08	48
Rural	20	All	0.96	0.62	1.47	12
Rural	20	All	0.88	0.65	1.18	24
Rural	20	All	0.92	0.77	1.10	48
Rural	50	All	1.05	0.52	2.11	12
Rural	50	All	0.87	0.51	1.48	24
Rural	50	All	0.84	0.59	1.20	48

B. Urban non-NYC Strata

Strata	Threshold (%)	Subgroup	OR	Lower	Upper	Window (hours)
Urban	10	All	1.11	0.95	1.31	12
Urban	10	All	1.06	0.98	1.16	24
Urban	10	All	1.03	0.98	1.08	48
Urban	20	All	1.20	0.96	1.49	12
Urban	20	All	1.12	1.00	1.26	24

Urban	20	All	1.05	0.98	1.12	48
Urban	50	All	1.88	1.05	3.36	12
Urban	50	All	1.52	1.08	2.15	24
Urban	50	All	1.18	0.99	1.39	48

C. NYC Strata

Strata	Threshold (%)	Subgroup	OR	Lower	Upper	Window (hours)
NYC	10	All	1.03	0.64	1.67	12
NYC	10	All	1.09	0.86	1.37	24
NYC	10	All	1.06	0.94	1.21	48
NYC	20	All				12
NYC	20	All	0.75	0.14	3.88	24
NYC	20	All	1.18	0.48	2.89	48
NYC	50	All				12
NYC	50	All				24
NYC	50	All				48

D. Pooled Strata

Strata	Threshold (%)	Subgroup	OR	Lower	Upper	Window (hours)
All	10	All	1.05	0.91	1.21	12
All	10	All	1.04	0.97	1.12	24
All	10	All	1.02	0.98	1.07	48
All	20	All	1.13	0.93	1.36	12
All	20	All	1.07	0.97	1.2	24
All	20	All	1.03	0.97	1.09	48
All	50	All	1.46	0.96	2.23	12

All	50	All	1.25	0.98	1.6	24
All	50	All	1.07	0.94	1.23	48
All	10	Burn	1.19	0.76	1.86	12
All	10	Burn	1.13	0.89	1.43	24
All	10	Burn	1.11	0.97	1.28	48
All	20	Burn	1.48	0.89	2.47	12
All	20	Burn	1.25	0.93	1.68	24
All	20	Burn	1.14	0.95	1.37	48
All	50	Burn	7.51	0.45	124.26	12
All	50	Burn	1.58	0.91	2.74	24
All	50	Burn	1.25	0.95	1.64	48
All	10	Fall	0.99	0.76	1.31	12
All	10	Fall	1.00	0.87	1.16	24
All	10	Fall	1.00	0.92	1.08	48
All	20	Fall	0.93	0.6	1.44	12
All	20	Fall	1.00	0.79	1.27	24
All	20	Fall	1.01	0.9	1.15	48
All	50	Fall	0.90	0.33	2.49	12
All	50	Fall	1.03	0.46	2.30	24
All	50	Fall	0.96	0.54	1.43	48
All	10	Struck	1.14	0.73	1.78	12
All	10	Struck	1.07	0.86	1.34	24
All	10	Struck	1.01	0.88	1.15	48
All	20	Struck	1.28	0.73	2.24	12
All	20	Struck	1.14	0.87	1.49	24
All	20	Struck	1.04	0.9	1.22	48

All	50	Struck				12
All	50	Struck	0.46	0.04	6.12	24
All	50	Struck	1.01	0.56	1.82	48
All	10	Transport	1.03	0.79	1.34	12
All	10	Transport	1.03	0.89	1.19	24
All	10	Transport	1.02	0.94	1.11	48
All	20	Transport	1.08	0.77	1.53	12
All	20	Transport	1.06	0.87	1.30	24
All	20	Transport	1.01	0.89	1.14	48
All	50	Transport	1.16	0.55	2.47	12
All	50	Transport	1.12	0.76	1.64	24
All	50	Transport	0.98	0.74	1.28	48
